# Pharmacometrics of 3-Methoxypterostilbene: A Component of Traditional Chinese Medicinal Plants

**DOI:** 10.1155/2013/261468

**Published:** 2013-04-16

**Authors:** Stephanie E. Martinez, Casey L. Sayre, Neal M. Davies

**Affiliations:** Faculty of Pharmacy, Apotex Centre University of Manitoba, Winnipeg, MB, Canada R3E 0T5

## Abstract

3-Methoxypterostilbene is a naturally occurring stilbene with potential in the treatment of diabetes. The preclinical pharmacokinetics and pharmacodynamics of 3-methoxypterostilbene were evaluated in the present study. The right jugular veins of male Sprague-Dawley rats were cannulated. The rats were dosed 10 mg/kg or 100 mg/kg of 3-methoxypterostilbene intravenously (IV) or orally (PO), respectively. Serum and urine samples were analyzed using a previously validated reversed-phase HPLC method. Serum AUC, serum *t*_1/2_, urine *t*_1/2_, Cl_total_, and Vd for IV dosing were 48.1 ± 23.8 **μ**g/h/mL, 18.9 ± 10.9 h, 9.54 ± 1.51 h, 47.8 ± 23.7 L/h/kg, and 5.11 ± 0.38 L/kg, respectively (mean ± SEM, *n* = 4) . Serum AUC, serum *t*_1/2_, urine *t*_1/2_, Cl_total_, and Vd for PO dosing were 229 ± 44.6 **μ**g/h/mL, 73.3 ± 8.91 h, 20.6 ± 3.01 h, 0.48 ± 0.008 L/h/kg, and 52.0 ± 10.5 L/kg, respectively (mean ± SEM, *n* = 4). Bioavailability of the stilbene was determined to be 50.6%  ± 10.0%. A 3-methoxypterostilbene glucuronidated metabolite was detected in both serum and urine. 3-Methoxypterostilbene exhibited antidiabetic activity including *α*-glucosidase and *α*-amylase inhibition as well as concentration-dependent antioxidant capacity similar to resveratrol. 3-Methoxypterostilbene also exhibited anti-inflammatory activity. 3-Methoxypterostilbene appears to be a bioactive compound and may be useful in reducing postprandial hyperglycemia.

## 1. Introduction

**Figure 1:**
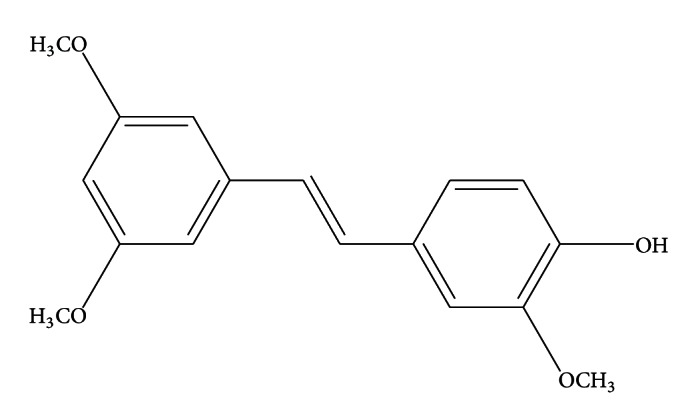
Chemical structure of 3-methoxypterostilbene.

3-Methoxypterostilbene (*trans*-3,3′-5-trimethoxy-4′-hydro-xystilbene), C_17_H_18_O_4_, MW 286.324 g/mol (Figure 1), is a naturally occurring stilbene [1, 2] that can also be easily synthesized using simple combinatorial synthesis [3–7]. 3-Methoxypterostilbene is a structural analogue of resveratrol, which has demonstrated a myriad of potential prohealth effects including anticancer, cardioprotective, anti-inflammatory, neuroprotective, and antiobesity properties [8]. However, it differs from resveratrol in its physicochemical properties. The predicted octanol water partition coefficient (XLogP) for 3-methoxypterostilbene is 3.54 ± 0.49 [9] and the experimentally determined XLogP for resveratrol is 1.53 ± 0.01 [10]. 

3-Methoxypterostilbene has been isolated in two plants used in traditional Chinese medicine. The compound has been found in *Sphaerophysa salsula *(also known as *Swainsona salsula*), a shrub called “ku ma du,” used for the treatment of hypertension [1]. 3-Methoxypterostilbene has also been found as an aglycone of a stilbene glycoside in the commonly used *Rheum palmatum* (Chinese rhubarb), called “da huang,” used to treat digestive disorders [2].

Due to 3-methoxypterostilbene's structural similarity to resveratrol and its presence in traditional Chinese medicinal plants, 3-methoxypterostilbene may also possess potential health benefits. However, information in the literature on the bioactivity of 3-methoxypterostilbene is scant. In a report seeking to identify biologically active piceatannol (another structural analogue of resveratrol) analogs with greater stability than piceatannol, 3-methoxypterostilbene was reported to demonstrate significantly greater activity in a 9 KB cytotoxicity assay as well as a crown-gall plant antitumor (potato disk) assay than piceatannol [3]. 3-Methoxypterostilbene proved to be as effective as resveratrol in the inhibition of bacterial lipopolysaccharide-induced tissue factor expression in human peripheral blood mononuclear cells in a study investigating resveratrol derivatives and coronary heart disease [11]. Several studies have also demonstrated that 3-methoxypterostilbene possesses greater apoptotic-inducing activity than resveratrol in human leukemia-derived HL60 cells [4, 12, 13].

With the ever-increasing natural products canon of knowledge and growing concern for obesity-related diseases, there has been much focus on the use of natural products to aid in the management and treatment of type 2 diabetes. Resveratrol has been extensively studied in animals for its potential to treat obesity and type 2 diabetes [8]. Resveratrol appears to be able to increase insulin sensitivity in various animal models of insulin resistance [14–18]. Additionally, animal studies using models that consisted of genetically obese rats and mice with dietary induced obesity or chemically induced diabetes found that resveratrol reduced blood glucose levels, which is important in the management of type 2 diabetes and prediabetic patients [14, 15, 19–25]. Thus, structural analogues of resveratrol may also possess antidiabetic properties similar to those of resveratrol. 

To our knowledge, there have been no studies evaluating the pharmacokinetics, disposition, or the *in vivo* metabolism of 3-methoxypterostilbene other than that of a single rat previously reported by Martinez et al. [9]. The objectives of the present study are to examine the pharmacokinetic disposition of 3-methoxypterostilbene as well as its *in vivo *metabolism and antidiabetic properties. To facilitate this, a reversed-phase high-performance liquid chromatographic (RP-HPLC) method was developed and validated for quantification of 3-methoxypterostilbene in biological matrices using ultraviolet detection [9]. Additionally, the objectives of this study were to investigate select biological activities of 3-methoxypterostilbene and perform a content analysis on commercially available dried traditional Chinese herbs reported to contain 3-methoxypterostilbene using the previously validated RP-HPLC method. For the first time, to our knowledge, the preclinical pharmacokinetics, antioxidant activity, cyclooxygenase-1 and -2 (COX-1 and -2) inhibition, and *α*-glucosidase and *α*-amylase inhibitory activity of 3-methoxypterostilbene are reported.

## 2. Materials and Methods

### 2.1. Chemicals and Reagents

3-Methoxypterostilbene was provided by the Sabinsa Corporation (Piscataway, NJ, USA) and pinosylvin was purchased from Sequoia Research Products Ltd. (Oxford, UK). HPLC-grade acetonitrile and water were purchased from J. T. Baker (Phillipsburg, NJ, USA). *β*-Glucuronidase type IX A (*β*-glucuronidase), poly(ethylene glycol) (PEG) 400, dimethyl sulfoxide (DMSO), *α*-glucosidase from *Saccharomyces cerevisiae*, 4-nitrophenyl *α*-D-glucopyranoside, 4-(2-hydroxyethyl)-1-piperazineethanesulfonic acid (HEPES), resveratrol, ibuprofen, etodolac, and *α*-amylase from porcine pancreas type VI-B were purchased from Sigma-Aldrich (St. Louis, MO, USA). Amylase HR reagent was purchased from Megazyme International Ireland (Wicklow, Ireland). *β*-Glucosidase from almonds was purchased from Tokyo Chemical Industry Co., Ltd. (Tokyo, Japan). Dried *Swainsona salsula* extract was provided by DaXingAnLing Snow Lotus Herb Bio-technology Co., Ltd. (Heilongjiang, China) and dried da huang (Chinese rhubarb) was purchased from Kwok Shing Ent. Ltd. (Scarborough, ON, Canada). The antioxidant activity kit and cyclooxygenase-1 and -2 inhibitor screening kits were purchased from Cayman Chemical Company (Ann Arbor, MI, USA). 

### 2.2. Chromatographic System and Conditions

The HPLC system used was a Shimadzu HPLC (Kyoto, Japan), consisting of two LC-10A pumps, a SIL-10AF autoinjector, a SPD-M10A photodiode array detector, and a SCL-10A system controller. Data collection and integration were achieved using Shimadzu EZ Start Class VP (version 7.4) software. A Phenomenex Luna C_18_(2) (5 *μ*m, 250 × 4.60 mm) column was used. The mobile phase consisted of acetonitrile and water (62 : 38 v/v) that were filtered and degassed under reduced pressure prior to use. Pinosylvin was used as the internal standard. Isocratic separation at ambient temperature using a flow rate of 1.05 mL/min was employed. Ultraviolet detection was carried out at 327 nm. Validation indicated that the precision of the assay was <12% (RSD) and was within 12% at the limit of quantification (LOQ) (0.05 *μ*g/mL). The bias of the assay was <15% and was within 13% at the LOQ [9].

### 2.3. Animals and Surgical Procedures

Male Sprague-Dawley rats (*≈*200 g) were obtained from Simonsen Laboratories (Gilroy, CA, USA) and allowed food (Purina Rat Chow 5001) and water *ad libitum* upon arrival to the vivarium. Rats were housed in a temperature- and humidity-controlled facility with a 12 h light/dark cycle. Prior to the first day of the pharmacokinetic experiment, the rats were anesthetized using isoflurane (IsoFlo, Abbott Laboratories, Abbot Park, IL, USA) coupled with an oxygen regulator, and monitored by pedal reflex and respiration rate to maintain a surgical plane of anesthesia. The right jugular veins of the rats were cannulated with sterile silastic cannulas (Dow Corning, Midland, MI, USA). After cannulation, Intramedic PE-50 polyethylene tubing (Becton, Dickinson and Company, Franklin Lakes, NJ, USA) was exposed through the dorsal skin. The cannulas were flushed with nonheparinized 0.9% sterile saline solution. The animals were placed in metabolic cages to recover and fasted overnight. 

Animal ethics approval was obtained from the University of Manitoba Office of Research Ethics and Compliance. 

### 2.4. Dosages

No pharmacokinetics studies are reported in the literature for 3-methoxypterostilbene. Despite the paucity of 3-methoxypterostilbene pharmacokinetic studies in the literature, studies on other stilbenes have reported intravenous (IV) dosage ranges from 10 to 20 mg/kg [26–30] and from 50 to 300 mg//kg for oral (PO) dosage [27, 31, 32]. In keeping with the literature, doses of 10 mg/kg IV and 100 mg/kg PO were chosen.

### 2.5. Pharmacokinetic Study

Male Sprague-Dawley rats (*n* = 8, average weight 200 g) were cannulated as described in the Animals and Surgical Procedures section. The animals were placed in metabolic cages following surgery where they were recovered overnight and fasted for 12 h prior to dosing. On the day of the experiment, the animals were dosed with 3-methoxypterostilbene in PEG 400 either IV (10 mg/kg, *n* = 4) or PO (100 mg/kg, *n* = 4). After dosing, a series of whole blood samples (0.5 mL) were collected at 0, 1, and 15 min, and 0.5, 1, 2, 4, 6, 12, 24, 48, and 72 h and 0, 0.25, 0.5, 1, 2, 4, 6, 12, 24, 48, and 72 h for IV and PO dosed rats, respectively. The cannulas were flushed with 0.5 mL 0.9% nonheparinized saline solution after each sample collection. The samples were collected into regular 2.0 mL Eppendorf tubes, centrifuged at 10,000 rpm for 5 min (Beckman Microfuge centrifuge, Beckman Coulter Inc., Fullerton, CA, USA), and the serum was collected. The serum was divided into two 0.1 mL fractions and placed into regular 2.0 mL Eppendorf tubes labeled as free and total serum samples. Samples were stored at −20°C until analyzed. Urine samples were also collected at 0, 2, 6, 12, 24, 48, and 72 h following 3-methoxypterostilbene administration. The volumes of urine produced by the rats were recorded, and two 0.1 mL aliquots were collected into separate prelabeled regular polypropylene microcentrifuge tubes and labeled as free and total urine samples and stored at −20°C until analyzed. At 72 h after dose, the animals were euthanized exsanguinated, and serum was collected.

### 2.6. Serum and Urine Sample Preparation

Serum and urine samples (0.1 mL) were run in duplicate with or without the addition of 20 *μ*L of 500 U/mL *β*-glucuronidase and incubated in a shaking water bath at 37°C for 2 h to liberate any glucuronide conjugates [33]. The proteins present in the serum samples were precipitated using 1 mL of −20°C acetonitrile. Urine and serum samples were vortexed (Vortex Genie-2, VWR Scientific, West Chester, PA, USA) for 30 s and centrifuged at 10,000 rpm for 5 min. The supernatants were transferred to new, labeled 2.0 mL Eppendorf microcentrifuge tubes. The samples were evaporated to dryness by a stream of nitrogen gas. The residues were reconstituted with 200 *μ*L of mobile phase, vortexed for 30 s, and centrifuged at 10,000 rpm for 5 min. The supernatants were transferred to HPLC vials, and 100 *μ*L was injected into the HPLC system for each sample.

*β*-Glucuronidase from *E*. *coli* type IX-A acts to specifically cleave the sugar moiety attached to the parent compound from the 3-methoxypterostilbene glucuronide back into the aglycone (3-methoxypterostilbene). The samples which did not undergo enzymatic hydrolysis (free samples) were utilized to determine the concentration of the aglycone, whereas the samples that did undergo enzymatic hydrolysis (total samples) were used to determine the concentration of the aglycone originally present in the sample in addition to the concentration of the glucuronidated metabolite cleaved back to 3-methoxypterostilbene. Therefore, by subtracting the free sample concentration from the total sample concentration, the concentration of the glucuronidated metabolite can be calculated without the use of an additional chromatographic run and analysis.

### 2.7. Pharmacokinetic Analysis

Pharmacokinetic analysis was performed using data from individual rats for which the mean and standard error of the mean (SEM) were calculated for each group. Samples were analyzed using WinNonlin software (version 1.0; Pharsight Corporation, Mountain View, CA, USA) to calculate the pharmacokinetic parameters. The rats' concentrations versus time points were subjected to a noncompartmental model. The apparent elimination rate constant (KE) was estimated from the slope of the log-linear phase of declining concentration versus time plot. The half-life and rate of eliminated were determined by applying the previously described software with the specified parameters. The renal clearance was calculated by multiplying the fraction of compound excreted unchanged with total body clearance. The plasma half-life (*t*_1/2_) was determined using the following equation: *t*_1/2_ = 0.693/KE. To determine the fraction of unchanged 3-methoxypterostilbene excreted (*f*_*e*_) in urine, the total amount of urine was divided by the total dose administered. The renal clearance (CL_renal_) was determined by the equation: CL_renal_ = *f*_*e*_ × CL_total_.

### 2.8. Content Analysis of Dried Traditional Chinese Medicinal Plants

3-Methoxypterostilbene has been reported to be present in two plants used in traditional Chinese medicine—*Sphaerophysa salsula *(*Swainsona salsula*) and *Rheum palmatum* (Chinese rhubarb) [1, 2]. Dried *S. salsula* extract and dried Chinese rhubarb are both commercially available products. Chinese rhubarb was frozen under liquid nitrogen and then ground into a fine powder. *S. salsula* extract was already in powdered form. Two 0.1 g of each product were measured and placed into 2.0 mL Eppendorf tubes. 1.5 mL methanol was added to each tube for extraction. Tubes were vortexed for 30 seconds, agitated for 3 h, and centrifuged at 10,000 rpm for 5 min. One of the duplicates was treated to extract only aglycones (free) and the second of the duplicates was treated to cleave any glycosides to aglycones (total) by using *β*-glucosidase from almonds. The supernatants from the free samples were transferred into new 2.0 mL Eppendorf tubes, and 50 *μ*L of internal standard, pinosylvin, was added. Samples were vortexed for 30 s, dried to completion under a stream of nitrogen gas, and stored at −20°C until analysis. The supernatants of the total samples were transferred to new 2.0 mL Eppendorf tubes, dried to completion under a stream of compressed nitrogen gas, and reconstituted with PBS (200 *μ*L at pH 7.4). 20 *μ*L of *β*-glucosidase (750 U/mL in PBS at pH 7.4) was added and samples were incubated for 48 h at 37°C in a shaking water bath. *β*-Glucuronidase acts by cleaving the glycosidic sugar moieties frequently present in plant extracts as previously described [29]. Acetonitrile (1 mL) was added to stop the enzymatic reaction, followed by the addition of internal standard (50 *μ*L). Samples were centrifuged at 10,000 rpm for 5 min and the supernatant was dried to completion under a stream of compressed nitrogen gas. Both free and total samples were reconstituted in mobile phase (200 *μ*L), and 100 *μ*L was injected into the HPLC under the same conditions previously described.

### 2.9. Antioxidant Capacity Determination

The antioxidant capacities of 3-methoxypterostilbene and resveratrol were measured through an assay that relied on the inhibition of the oxidation of ABTS (2,2′-azino-di-[3-ethylbenzthiazoline sulphonate]) to ABTS^*∙*+^ by metmyoglobin. The amount of ABTS^*∙*+^ can be monitored spectrophotometrically. The degree of suppression of absorbance caused by the compound of interest is proportional to the concentration of ABTS^*∙*+^, which is expressed as Trolox equivalents (*μ*g/mL). For this assay, 3-methoxypterostilbene and resveratrol were dissolved in DMSO on the day of the experiment to yield concentrations of 1, 5, 10, 50, and 100 *μ*g/mL. No additional dilution was employed. To run the assay, 10 *μ*L of sample was combined with 10 *μ*L metmyoglobin and 150 *μ*L chromogen. Then, 40 *μ*L of hydrogen peroxide working solution was added within 1 min to all the samples. The plate was covered and incubated on a shaker for 5 min at room temperature 923 ± 1°C), and the absorbance was measured at 750 nm using the Synergy HT multiwell plate reader and Gen5 data analysis software (Biotek Instruments Inc., Winooski, VT, USA). The assay was performed in quadruplet. For more information regarding the assay protocol, please refer to the instructions for the kit (Antioxidant Assay Kit from Cayman Chemical—Cat. no. 709091). 

### 2.10. Cyclooxygenase Inhibition Determination

The inhibition of COX-1 and -2 by 3-methoxypterostilbene was measured through the use of a three-day commercial assay kit. The assay relied on the quantification of the prostanoid product based on enzyme immunoassay (EIA), which uses a nonspecific antibody for prostaglandins (PGs). The constant concentration of the PG-AChE-tracer and the varying concentrations of the PGs available to bind to the antiserum is inversely proportional to the concentration of free PGs in the well. For this assay, 3-methoxypterostilbene was dissolved in DMSO over the concentration range of 1–250 *μ*g/mL. Ibuprofen and etodolac, COX-2 preferential NSAIDs, were dissolved in DMSO and used as controls over the concentration range of 1–250 *μ*g/mL. Reagents were prepared according to the manufacturer's instructions that accompanied the kit. The ELISA plate for this purpose was read at an absorbance of 415 nm within 10 minutes at room temperature (23 ± 1°C) using the Synergy HT multiwell plate reader and Gen5 data analysis software (Biotek Instruments Inc., Winooski, VT, USA). The assay was performed in quadruplet. For more information regarding the assay protocol, please refer to the instructions for the kit (COX Inhibitor Screening Assay Kit from Cayman Chemical—Cat. no. 560131).

### 2.11. *α*-Glucosidase Inhibition Determination

Inhibition of *α*-glucosidase was determined through a colorimetric assay adapted and modified from the method presented by Tadera et al. [34]. The assay uses *p-*nitrophenyl-*α*-D-glucopyranoside (PNPG), which is hydrolyzed specifically by *α*-glucosidase into a yellow-colored product (*p*-nitrophenol). The absorbance at 410 nm of liberated *p-*nitrophenol was measured. For this assay, 3-methoxypterostilbene and resveratrol were dissolved in DMSO to create concentration ranges from 0 to 200 *μ*g/mL. 160 *μ*L of 100 mM phosphate buffer (pH 6.8), 25 *μ*L of 20 mM PNPG in phosphate buffer, and 10 *μ*L of stilbenes in DMSO were added to wells of a 96-well plate (10 *μ*L DMSO was added to the control wells). The plate was incubated at 30°C for 5 min and then 10 *μ*L of the buffer containing 0.02 mg/mL of enzyme was added to each well. The plate was further incubated for 5 min. 20 *μ*L of 3.25 M sodium hydroxide was added to each well to stop the reaction. The plate was immediately read at an absorbance of 410 nm at room temperature (23 ± 1°C) using the Synergy HT multiwell plate reader and Gen5 data analysis software (Biotek Instruments Inc., Winooski, VT, USA). The assay was performed in sextuplicate. 

Inhibition (%) was calculated as ((*A* − *B*)/*A*) × 100, where *A* was the average absorbance of the control wells and *B* was the absorbance of the wells containing stilbenes.

### 2.12. Alpha-Amylase Inhibition Determination

Inhibition of *α*-amylase was determined through a colorimetric assay also adapted and modified from the method presented by Tadera et al. [34]. A synthetic substrate, nonreducing-end-blocked *p*-nitrophenyl maltoheptaoside (BPNPG7) commercially prepared as amylase HR reagent, which is hydrolyzed specifically by *α*-amylase into *p*-nitrophenyl maltosaccharide is employed. The *α*-glucosidase present in the assay then converts the new substrate into *p*-nitrophenol and absorbance at 410 nm is measured as previously stated in the *α*-amylase assay. 3-methoxypterostilbene and resveratrol were dissolved in methanol to create concentration ranges from 0 to 200 *μ*g/mL. 100 *μ*L of amylase HR reagent (prepared following directions accompanying the reagent, Megazyme Amylase HR Reagent, Cat. no. R-AMHR4), and 40 *μ*L of stilbene in methanol were added to a 96-well plate (40 *μ*L of methanol was added to the control wells). The plate was incubated for 5 minutes at 37°C and then 60 *μ*L of 0.1 mg/mL *α*-amylase in 0.1 M HEPES buffer (pH 6.9) was added to the reaction mixture. After further incubation at 37°C, for 10 min, 20 *μ*L of 3.25 M sodium hydroxide was added to each well to stop the reaction. The liberated *p*-nitrophenol was determined and the percent inhibition calculated as described in the assay for *α*-glucosidase activity previously described. The assay was performed in sextuplicate.

### 2.13. Statistical Analysis

Compiled data were presented as mean and standard error of the mean (mean ± SEM). Where possible, the data were analyzed for statistical significance using Minitab 15 statistical software (Minitab, Inc., State College, PA, USA). Student's *t*-test was employed for unpaired samples with a value of *P* < 0.05 being considered statistically significant. 

## 3. Results and Discussion

### 3.1. Pharmacokinetic Study

Standard curves established linearity over the concentration range studied for the serum and urine samples. Chromatograms showed no interference from endogenous components. Total samples (incubated with *β*-glucuronidase from *Escherichia coli* type IX-A) verified the presence of a glucuronidated metabolite based on the increase in 3-methoxypterostilbene (aglycone parent compound) concentrations after enzymatic hydrolysis in both serum and urine. Glucuronidation of 3-methoxypterostilbene parallels previous rat and human studies with resveratrol existing predominately in its conjugated form in both plasma and urine [35]. 

**Figure 2:**
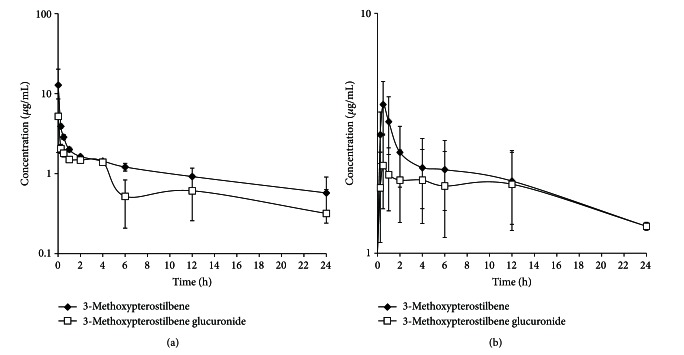
(a) 3-Methoxypterostilbene disposition in serum following intravenous administration. (b) 3-Methoxypterostilbene disposition in serum following oral administration (*n* = 4, mean ± SEM).

The serum concentration versus time profile for IV-dosed 3-methoxypterostilbene demonstrates a rapid decline in concentration in the first hour, representing a distribution phase, which was followed by a steady elimination up to 24 hours, after which the serum concentrations were below detectable concentrations (0.05 *μ*g/mL) (Figure 2(a)). 3-Methoxypterostilbene dosed PO displayed rapid absorption with an average *T*_max⁡_ of 30 minutes (Figure 2(b)). The glucuronidated metabolite in both routes of administration appeared to display multiple peaking which is suggestive of enterohepatic recycling as indicated by an increase in serum concentration around 4 h after dose. Enterohepatic recycling has previously been reported for resveratrol [27, 36].

Table 1 summarizes the pharmacokinetic parameters exhibited by 3-methoxypterostilbene at an IV dose of 10 mg/kg and a PO dose of 100 mg/kg. Noncompartmental analysis in WinNonlin software (ver. 1.0) was used to model both serum and urine data. The total serum clearance of 3-methoxypterostilbene was determined to be 47.8 ± 23.7 L/h/kg for IV dosing and 0.480 ± 0.0800 for PO dosing. The mean fraction excreted in urine unchanged (*f*_*e*_) was 1.64 ± 0.950% for IV and 1.24 ± 0.160% for PO, indicating that 3-methoxypterostilbene is mainly excreted via nonrenal routes. Renal clearance (CL_renal_) was measured at 0.760 ± 0.46 L/h/kg for IV and 0.0100 ± 0.00100 L/h/kg for PO, and hepatic clearance (CL_hepatic_ = CL_total_ − CL_renal_) was determined to be 47.1 ± 23.3 L/h/kg for IV and 0.480 ± 9.0800 L/h/kg for PO assuming that nonrenal clearance is hepatic clearance. The volume of distribution of 3-methoxypterostilbene is 5.11 ± 0.380 L/kg IV and 52.0 ± 10.0 L/kg PO, which is greater than total body water, suggesting that 3-methoxypterostilbene is highly distributed into tissues. The mean area under the curve (AUC), representing the total amount of exposure in the serum over time, was 48.1 ± 23.8 *μ*g·h/mL for IV and 229 ± 44.6 *μ*g·h/mL for PO. The serum concentration of 3-methoxypterostilbene declined very slowly with a mean elimination half-life of 18.9 ± 10.9 h for IV and 73.3 ± 8.91 h for PO. The oral bioavailability for 3-methoxypterostilbene was determined to be 50.6%0020  ± 10.0%.

Reported bioavailability of resveratrol in rats ranges from 20 to 38.8% [27, 32] and <1% in humans [37] with a wide variability in pharmacokinetic parameters among individuals [38]. Species-dependent rapid conjugation with higher glucuronidation rates and affinity in humans may limit stilbene bioavailability and show expressed differences in pharmacokinetics between rodent and human studies. Poor bioavailability in humans is a potential limitation in the use of resveratrol as a therapeutic agent, hence the interest in structural analogs. 3-Methoxypterostilbene bioavailability in rats has been determined to be 50.6% ± 10.0%. The melting point range of 3-methoxypterostilbene was experimentally determined to be 88.5–91.2°C. The reported melting point range of resveratrol is 253–255°C [39]. Therefore, 3-methoxypterostilbene has a lower crystallinity than resveratrol. The low crystallinity of 3-methoxypterostilbene is responsible for its increased dissolution over resveratrol. Furthermore, the aqueous solubility of resveratrol is ~30 mg/mL [40] and the predicted aqueous solubility, using AlogPs [41] of 3-methoxypterostilbene is 8–88 mg/L. The increased bioavailability of 3-methoxypterostilbene in rats compared to resveratrol is likely due to its lower crystallinity resulting in enhanced dissolution over resveratrol and may be in part due to differences in solubility and absorbance mechanisms which will be examined in further studies. The increased bioavailability of 3-methoxypterostilbene over resveratrol in rats may extend to the greater bioavailability of 3-methoxypterostilbene than that of resveratrol in humans. 

**Figure 3:**
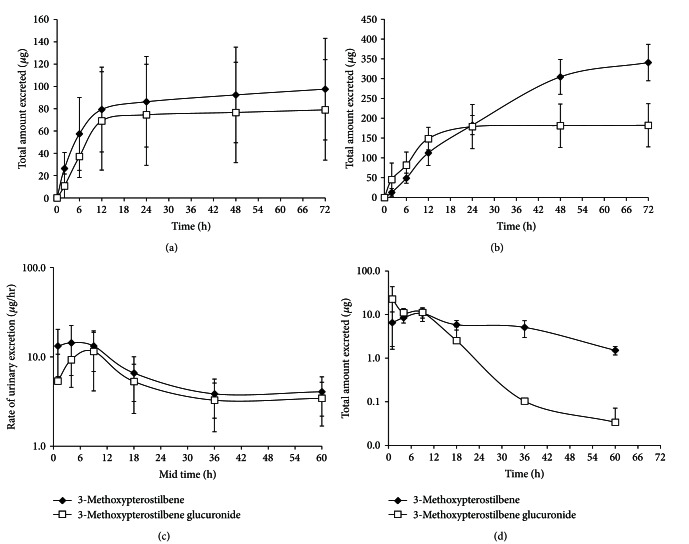
(a) Cumulative 3-methoxypterostilbene and glucuronidated metabolite (*μ*g) excreted in urine over 72 h after intravenous administration. (b) Cumulative 3-methoxypterostilbene and glucuronidated metabolite (*μ*g) excreted in urine over 72 h after oral administration. (c) Rate of excretion (*μ*g/h) of 3-methoxypterostilbene and glucuronidated metabolite in urine over 72 h after intravenous administration. (d) Rate of excretion (*μ*g/h) of 3-methoxypterostilbene and glucuronidated metabolite in urine over 72 h after oral administration (*n* = 4 mean ± SEM).

Analysis of urine samples for both routes of administration displayed the presence of the parent compound, 3-methoxypterostilbene and the glucuronidated metabolite previously identified in the serum (Figures 3(a) and 3(b)). The total cumulative urinary excretion plot (Figure 3(a)) indicates that 3-methoxypterostilbene is excreted predominantly in the aglycone form for PO administration and almost equally in the aglycone and glucuronidated metabolite form for IV dosing. The glucuronidated metabolite appears to be mostly excreted by 12 h after dose for both routes of administration while 3-methoxypterostilbene (aglycone) appeared to be predominately excreted by 12 h after dose for IV but steadily increased in excretion even at 72 h after dose for PO administration. The half-life of 3-methoxypterostilbene in urine was determined to be 9.54 ± 1.41 h for IV and 20.6 ± 3.01 h for PO. The rate of urinary excretion plot (Figure 3(b)) indicates that 3-methoxypterostilbene and its glucuronidated metabolite have similar rates of excretion as indicated by their parallel slope (−KE/2.303) for IV administration but the glucuronidated metabolite appears to have a greater rate of excretion over the aglycone after PO administration.

The total dose of 3-methoxypterostilbene administered was 10 mg/kg for IV and 100 mg/kg PO. The average weight of the rats in this experiment was ~200 g. Each rat received ~2 mg of 3-methoxypterostilbene IV and 20 mg PO. The plots of cumulative amount excreted in urine for both the aglycone and glucuronidated metabolite forms excreted (~98 *μ*g and 80 *μ*g, resp., for IV and 341 *μ*g and 182 *μ*g, resp., for PO) are very small compared to the overall dose administered (~2 mg and 20 mg for IV and PO, resp.). This further suggests that 3-methoxypterostilbene is eliminated predominately by nonrenal routes. As previously mentioned, *f*_*e*_ was 1.64 ± 0.950% for IV and 1.24 ± 0.160% for PO, and therefore CL_renal_ was 0.760 ± 0.460 L/h/kg for IV and 0.0100 ± 0.00100 L/h/kg for PO. Excretion via nonrenal routes for 3-methoxypterostilbene agrees with literature reports of nonrenal excretion of other stilbenes [27, 30].

### 3.2. Content Analysis of Dried Traditional Chinese Medicinal Plants

Evaluation of the *S. salsula* extract and dried *R. palmatum* indicated that only the *S. salsula* extract contained detectable levels of 3-methoxypterostilbene. The aglycone concentration for the *S. salsula *extract was determined to be 0.842 *μ*g/g and the total concentration of 3-methoxypterostilbene (aglycone and glycoside) was determined to be 0.853 *μ*g/g indicating that 3-methoxypterostilbene exists primarily in its aglycone form in *S. salsula* extract. Despite the report that 3-methoxypterostilbene exists as an aglycone of a stilbene glycoside in* R. palmatum* [2], the compound was not detected as an aglycone or glycoside in the commercially available dried Chinese rhubarb. It is suspected that 3-methoxypterostilbene may be detectable in other commercially available Chinese rhubarb samples and that plant variation likely accounts for the lack of detection in this sample. 

### 3.3. Antioxidant Capacity of 3-Methoxypterostilbene

**Figure 4:**
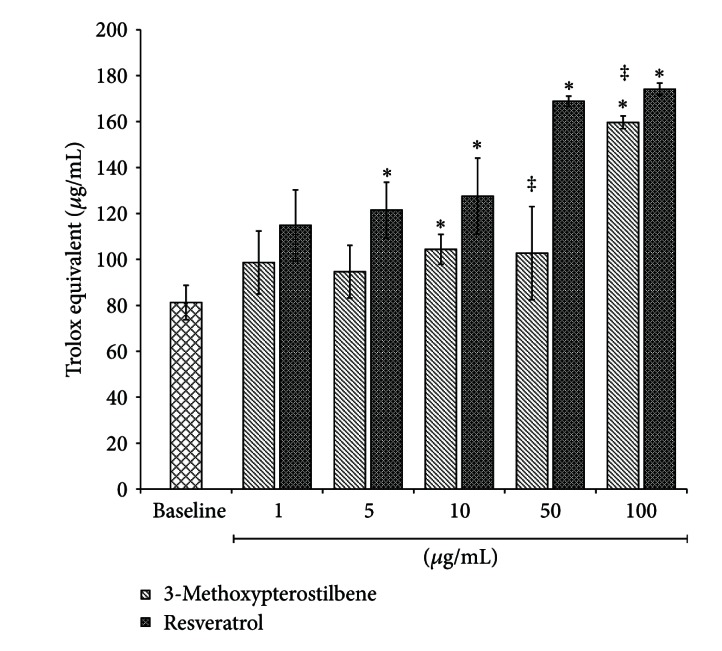
Antioxidant capacity (*n* = 4, mean ± SEM) of 3-methoxypterostilbene and resveratrol at 1, 10, 50, and 100 *μ*g/mL dissolved in DMSO. *Significantly greater than baseline (DMSO) sample (*P* < 0.05). ^‡^Significantly different from resveratrol at the same concentration (*P* < 0.05).

Figure 4 reports the antioxidant capacity of 3-methoxypterostilbene in units of Trolox equivalents (*μ*g/mL). The baseline (DMSO only) samples have a low antioxidant capacity (81.2 ± 7.47 *μ*g/mL or 0.325 ± 0.0299 mM). 3-Methoxypterostilbene demonstrates a modest concentration-dependent antioxidant activity with an antioxidant capacity at 1 *μ*g/mL of 98.6 ± 13.7 *μ*g/mL (0.394 ± 0.0546 mM) Trolox equivalents and a capacity at 100 *μ*g/mL of 174 ± 2.70 *μ*g/mL (0.695 ± 0.00940 mM) Trolox equivalents. This indicated that 3-methoxypterostilbene prevents oxidation at comparable levels to Trolox, if not better (as seen at lower concentrations). 3-Methoxypterostilbene demonstrated significantly greater activity to the baseline samples from 10 to 100 *μ*g/mL (*P* < 0.05) and only showed significant difference from resveratrol activity at the two highest concentrations tested, 50 and 100 *μ*g/mL (*P* < 0.05).

The ability of 3-methxoypterostilbene to work as an antioxidant is of paramount importance as the wide range of health benefits of polyphenols are thought to result at least in part from their antioxidant capacity. For example, the ability of resveratrol to limit the start and progression of atherosclerosis is associated with the compound's ability to inhibit lipid oxidation of polyunsaturated fatty acids [42]. It is likely that the health benefits of 3-methoxypterostilbene will also be associated with its antioxidant capacity. In the literature, it is well known that the number and position of the hydroxyl groups on stilbenes are critical for bioactivity and *t* antioxidant activity [43–46]. A computer model of antioxidant activity of hydroxystilbenes suggests that 3-methoxypterostilbene is one of the most potent 3,4 hydroxystilbenes modeled and has greater potency than resveratrol [47].

### 3.4. Cyclooxygenase Inhibitory Activity

**Figure 5:**
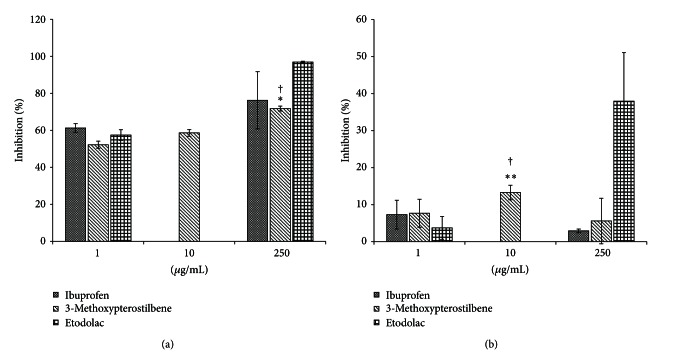
(a) Cycloxoygenase I inhibition activity of 3-methoxypterostilbene at 1, 10, and 250 *μ*g/mL. (b) Cycloxoygenase II inhibition activity of 3-methoxypterostilbene at 1, 10, and 250 *μ*g/mL. *Significantly greater activity than 1 *μ*g/mL ibuprofen (*P* < 0.05). **Significantly greater activity than 250 *μ*g/mL ibuprofen (*P* < 0.05). ^†^Significantly greater activity than 1 *μ*g/mL etodolac (*P* < 0.05). ^‡^Significantly greater activity than 250 *μ*g/mL etodolac (*P* < 0.05) (*n* = 4, mean ± SEM).

Figure 5 reports the cyclooxygenase inhibitory activity of 3-methoxypterostilbene and two NSAIDs: etodolac and ibuprofen. 3-Methoxypterostilbene appears to have greater activity against COX-1 than COX-2.  Figure 5(a) demonstrates the positive concentration-dependent inhibitory activity of 3-methoxypterostilbene against COX-1. While 3-methoxypterostilbene did not demonstrate significantly greater (*P* < 0.05) COX-1 inhibition activity than the two NSAIDs at lower concentrations, the high concentration (250 *μ*g/mL) of 3-methoxypterostilbene demonstrated significantly greater activity (*P* < 0.05) than the NSAIDs at low doses.  Figure 5(a) details the COX-2 inhibitory activity of 3-methoxypterostilbene. At the concentration of 10 *μ*g/mL, 3-methoxypterostilbene demonstrated statistically greater inhibition (*P* < 0.05) than etodolac at 1 *μ*g/mL and ibuprofen at 250 *μ*g/mL. Several stilbenes, including resveratrol, are known to be preferential COX-2 inhibitors [28]. 

### 3.5. Antidiabetic Activity

#### 3.5.1. Alpha-Glucosidase Activity

**Figure 6:**
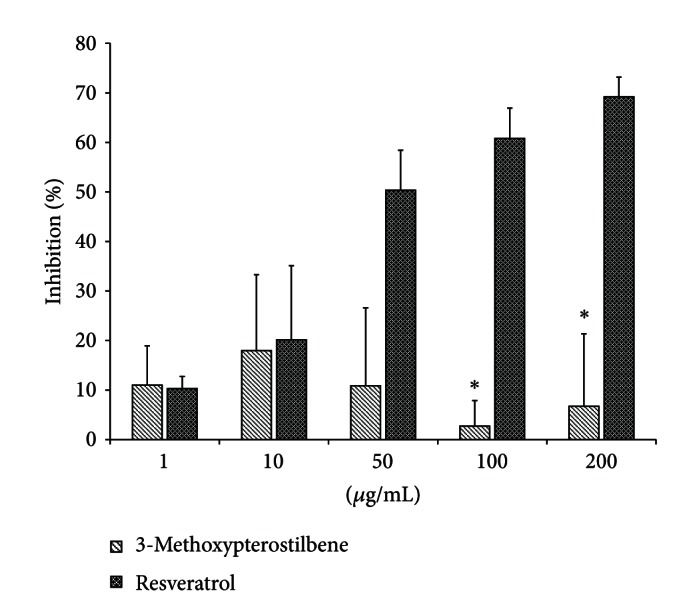
Alpha-glucosidase inhibition activity of 3-methoxypterostilbene and resveratrol (*n* = 6, mean ± SEM). *Significantly different from resveratrol at the same concentration (*P* < 0.05).

Figure 6 reports the alpha-glucosidase inhibition of 3-methoxypterostilbene and resveratrol. Resveratrol shows a clear positive concentration-dependent inhibition relationship whereas 3-methoxypterostilbene does not appear to exhibit greater inhibition at higher concentrations. *α*-Glucosidase inhibition activity is only statistically different (*P* < 0.05) between the two stilbenes at the two highest concentrations tested (100 and 200 *μ*g/mL). 

*α*-Glucosidase is an enzyme found in the small intestine which hydrolyzes 1,4-*α*-bonds of disaccharides into glucose. Inhibition of *α*-glucosidase suppresses postprandial hyperglycemia by lowering the rate of glucose absorption via delayed carbohydrate digestion and extended digestion time. *α*-Glucosidase inhibitors are useful in maintaining glycemic control of prediabetic and type two diabetic patients. The lower concentrations of 3-methoxypterostilbene tested may be biologically achievable resulting in moderate inhibition of *α*-glucosidase comparable to that of resveratrol, and reduction of postprandial hyperglycemia could be seen.

#### 3.5.2. Alpha-Amylase Activity

**Figure 7:**
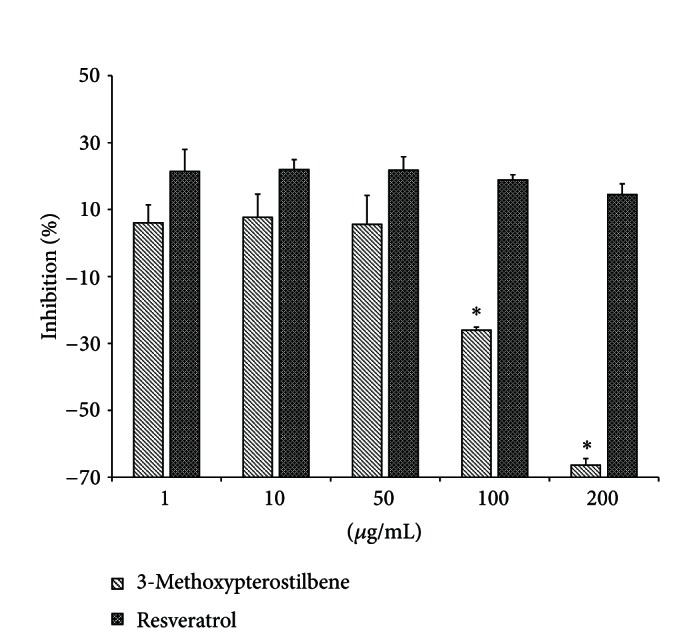
Alpha-amylase inhibition activity of 3-methoxypterostilbene and resveratrol (*n* = 6, mean ± SEM). *Significantly different from resveratrol at the same concentration (*P* < 0.05).

*α*-Amylase inhibition activity by 3-methoxypterostilbene and resveratrol is shown in Figure 7. Both resveratrol and 3-methoxypterostilbene display relatively weak inhibition of *α*-amylase, and both stilbenes show a slightly negative concentration inhibition relationship. At the highest concentrations tested, 3-methoxypterostilbene appears to increase activity of *α*-amylase. At lower, biologically relevant concentrations (1-50 *μ*g/mL), there is no significant difference between inhibition activity of 3-methoxypterostilbene and resveratrol. 

In humans, *α*-amylase is an enzyme predominately found in the pancreas and saliva. Like *α*-glucosidase, *α*-amylase hydrolyzes *α*-1,4-glycosidic bonds but acts on polysaccharides. Inhibition of *α*-amylase also reduces postprandial hyperglycemia, and *α*-glucosidase inhibitors may be used to treat type 2 diabetes. The modest inhibitory activity of 3-methoxypterostilbene at biologically relevant levels suggests that it may be as effective as resveratrol at helping reduce postprandial hyperglycemia. 

## 4. Conclusions

In summary, the pharmacokinetics in rats and the *in vitro* metabolism of 3-methoxypterostilbene was evaluated for the first time. 3-methoxypterostilbene demonstrates improved bioavailability compared to resveratrol. 3-Methoxypterostilbene demonstrated antioxidant activity comparable to resveratrol at biologically relevant concentrations. This stilbene also showed strong COX-1 inhibition comparable to two NSAIDs and moderate inhibition of COX-2. 3-Methoxypterostilbene demonstrated moderate antidiabetic activity via inhibition of *α*-glucosidase and *α*-amylase comparable to resveratrol. Further exploration of the pharmacodynamics of 3-methoxypterostilbene is under way to demonstrate utility in reducing postprandial hyperglycemia, adiposeness, cardiac hypertrophy and inflammation.

## Figures and Tables

**Table 1:** Pharmacokinetic parameters of 3-methoxypterostilbene in the rat.

Pharmacokinetic parameter	Intravenous Mean ± SEM	Oral Mean ± SEM
AUC_inf_ (*µ*g·h/mL)	48.1 ± 23.8	229 ± 44.6
Vd_*β*_ (L/kg)	5.11 ± 0.380	52.0 ± 10.5
CL_hepatic_ (L/h/kg)	47.1 ± 23.3	0.480 ± 0.0800
CL_renal_ (L/h/kg)	0.760 ± 0.460	0.0100 ± 0.000
CL_total_ (L/h/kg)	47.8 ± 23.7	0.480 ± 0.0800
*f*_*e*_ (%)	1.64 ± 0.950	1.24 ± 0.160
*t*_1/2_ (h) serum	18.9 ± 10.9	73.3 ± 8.91
*t*_1/2_ (h) urine	9.54 ± 1.51	20.6 ± 3.01
MRT (h)	26.0 ± 15.0	105 ± 13.1
Bioavailability (F%)	100	50.6 ± 10.0
